# Predictive role of systemic immune-inflammation index and neutrophil/lymphocyte ratio values in infants with retinopathy of prematurity

**DOI:** 10.1007/s00417-024-06493-y

**Published:** 2024-04-24

**Authors:** Oğuzhan Oruz, Mehmet Serdar Dervişoğulları, Müzeyyen Ezgi Öktem, Caner İncekaş

**Affiliations:** 1 Department of Ophthalmology, Başkent University School of Medicine, Adana, Turkey; 2 Department of Ophthalmology, Adana City Hospital, Adana, Turkey; 3 Department of Biostatistics, Baskent University Faculty of Medicine, Ankara, Turkey

**Keywords:** Systemic immune-inflammation index, Prematurity of retinopathy, Neutrophile/lymphocyte ratio, Platelet/lymphocyte ratio, Lymphocyte/monocyte ratio

## Abstract

**Purpose:**

To search the relationship between serum neutrophil-to-lymphocyte ratio (NLR), platelet-to-lymphocyte ratio (PLR), lymphocyte-to-monocyte ratio (LMR), and systemic immune-inflammation index (SII) values with the development of retinopathy of prematurity (ROP) and the requirement for laser treatment.

**Methods:**

This retrospective cohort study was carried out with 195 preterm infants between 2012 and 2023. The NLR, PLR, LMR, and SII values were calculated on both the first day and at the end of the first month after birth. The association between development of ROP and other risk factors were analyzed using univariate analysis and multivariate logistic regression analysis.

**Results:**

Of patients, 92 infants were diagnosed with ROP. Laser treatment was administered to 36 infants. The postnatal first-day NLR and SII values were higher in infants with ROP than in infants without ROP (p < 0.001 for both). Postnatal first-month NLR, LMR, and SII values were higher in infants with ROP (p < 0.001, p = 0.007, and p < 0.001, respectively). In multivariate analyses, postnatal first-day NLR and first-month LMR values were regarded as independent risk factors for the development of ROP (OR:8.867 and 1.286, p = 0.002 and p = 0.009, respectively). In multivariate analyses performed for laser treatment requirement, postnatal first-month PLR and SII values were determined as independent risk factors (OR:0.951 and 1.011, respectively, p = 0.004 for both).

**Conclusions:**

Postnatal first-day NLR and first-month LMR values were determined as independent risk factors for the development of ROP. For the requirement of laser treatment, the postnatal first-month PLR and SII values were determined as independent risk factors.


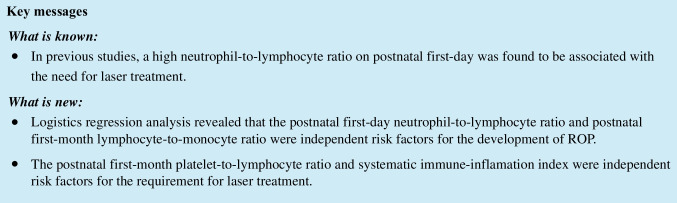


## Introduction

Despite many advances in neonatal care, Retinopathy of Prematurity (ROP) leads to low visual acuity [1]. The increase in the survival rate of preterm neonates together with the development of intensive care facilities has yielded an increase in the frequency of ROP [2]. Although ROP is a multifactorial disease, low birth weight and gestational age (GA) are the two most important risk factors [3]. Respiratory distress syndrome (RDS), surfactant treatment, number of days on the incubator, patent ductus arteriosus (PDA) and multiple pregnancy are other risk factors [3–5].

Systemic inflammation may impair angiogenesis, and the ensuing changes in oxygen saturation can lead to retinal ischemia [6]. There are two phases of ROP development. In the first phase, retinal vascularization is disrupted by hyperoxia, and in the second phase, abnormal vascularization is observed because of cytokine release due to hypoxia [7].

In previous studies, increased white blood cell subtype levels have been associated with inflammation [8]. Neutrophil–lymphocyte ratio (NLR), platelet-lymphocyte ratio (PLR), lymphocyte monocyte ratio (LMR), and systemic immune-inflammation index (SII) have been investigated as potential biomarkers of inflammation in terms of diagnosis and prognosis of many diseases such as cancers, cardiovascular and rheumatological diseases [9–11]. Moreover, in ophthalmology, such diseases as age-related macular degeneration, glaucoma, and diabetic retinopathy have also been scrutinized [12–14].

Although NLR has been studied many times in ROP [15, 16], there are only a limited number of studies focusing on the clinical significance of the PLR, LMR, and SII [17]. Given the role of inflammation in the pathogenesis of ROP, it is important to investigate the differences between the SII, NLR, PLR, and LMR in infants with and without ROP to determine the development and prognosis of ROP.

## Methods

This retrospective-cohort study included patients who were screened for ROP in the Başkent University Neonatal Intensive Care Unit from 2012 to 2023. The study was conducted in accordance with the tenets of the Declaration of Helsinki and was approved by the Başkent University Institutional Review Board (Project no: KA23/181) and supported by the Başkent University Research Fund. Informed consent was obtained from the parents of all infants.

İnfants with a GA of < 32 weeks and a birth weight (BW) of < 1500 g were included in the study. However, infants with a GA of > 32 weeks and BW of > 1500 g, for whom ROP screening was recommended by the neonatologist, were also screened. Neonates with hematological disease, sepsis, necrotizing enterocolitis, postnatal steroid therapy or blood product transfusion were excluded from the study. In addition, we did not include patients in whom intravitreal anti-VEGF was administered because of the small number of patients.

ROP screening was performed by an ophthalmologist experienced in diagnosis and treatment, and the International Classification of ROP (ICROP) guidelines was used to record the stage of the disease, location by zone, and signs of plus disease [18]. After pupil dilation with two drops of tropicamide 0.5% and phenylephrine 2.5% at 10-min intervals, the examination process was performed with an indirect ophthalmoscope using a 20/28 D lens. The first examination of premature infants was carried out between postnatal fourth and sixth weeks. Early Treatment for Retinopathy of Prematurity (ETROP) was followed as the treatment criteria [19]. Laser photocoagulation was performed in patients who met the treatment criteria. These criteria included zone 1 ROP with plus disease or zone 1 stage 3 ROP without plus disease or zone 2 stage 2 or 3 ROP with plus disease. Ophthalmological examinations continued until retinal vascularization reached the ora serrata. Data was recorded for the most advanced stage of ROP in the eye.

CBC results on the postnatal first-day and at the end of the first-month were obtained from medical records. CBC was calculated using the CELL-DYN Ruby (Abbott Laboratories, Diagnostic Division, Abbott Park, IL, USA) device. NLR was calculated by dividing the absolute neutrophil count by the absolute lymphocyte count, PLR by dividing the absolute platelet count by the absolute lymphocyte count, and LMR by dividing the absolute lymphocyte count by the absolute monocyte count. SII was calculated using the neutrophil × platelet/lymphocyte formula. The GA, BW, sex, and daily weight gain (DWG), the number of days in the incubator, DWG, the number of days with invasive and non-invasive oxygen intake, RDS, surfactant therapy, multiple pregnancy, and PDA were reported as risk factors.

## Statistical analysis

The minimum sample size was determined to be 64 infants with ROP and 64 without ROP, which would provide 80% test power at a 95% confidence level with an effect size of 0.5. For logistic regression analysis, the number of patients who provided 80% test power at the 95% confidence level, with an odds ratio of 0.55, was found to be 147. Statistical analyses were performed using SPSS software (version 25.0). The conformity of the variables to normal distribution was analysed by Shapiro–Wilk test. The mean, standard deviation, median, minimum, and maximum values were used for descriptive analyses. Independent Sample t-Test was used to determine the normally distributed variables between two groups. Frequency and percentage values of the variables were used in order to present the categorical variables. The relationships between categorical variables were analysed by Fisher-Freeman-Halton Exact Test. Differences between groups were determined by Dunn's Bonferroni Test. Variables with a significance value of p < 0.20 as a result of univariate logistic regression analyses were included in a multivariate logistic regression model to identify the factors that increased the risk of laser treatment requirements and ROP development. Forward and backward selection methods were adopted to obtain the best model in multivariate logistic regression analysis. In order to determine the threshold value in terms of the measurements, ROC analysis was used. The cases where the p-value was below 0.05 were considered statistically significant.

## Results

Data from 2317 patients were obtained from medical records, with 195 patients meeting the inclusion criteria. Of these, 98 (50.3%) were male. ROP was detected in 92 (47.2%) patients. Among infants, 42 (46.7%) had stage 1, 14 (15.2%) had stage 2, and 36 (39.1%) had stage 3 ROP. Stage 4 and 5 ROP were not observed. ROP was diagnosed at a mean of 34,47 ± 1,84 weeks (range, 30–40). Plus disease was observed in 40 (43.5%) infants. Laser photocoagulation was applied to 36 (39.1%) infants. A summary of the demographic data and clinical characteristics is provided in Table 1.

**Table 1:** Demographic data and clinical characteristics of patients

Variables			Total (n = 195)	ROP group (n = 92)	Non-ROP group (n = 103)	P^1^ value	ROP with laser (n = 36)	ROP without laser (n = 56)	P^2^ value
Gender		Male	98 (50.3%)	48 (52.17)	50 (48.54)	0.613	19(52.78%)	29(51.79%)	0.926
		Female	97 (49.7%)	44 (47.83)	53 (51.46)		17(47.22%)	27(48.21)	
Gestational age (weeks)			28.99 ± 2.24 (24–34)	27.61 ± 1.65 (24–33)	30.22 ± 1.97 (26–34)	** < 0.001**	26.92 ± 1.52 (24–30)	28.05 ± 1.59 (25–33)	** < 0.001**
Birth wieght (g)			1240 ± 313.80 (690–1960)	1087.17 ± 272.98(700–1940)	1376.5 ± 284.47 (690–1960)	** < 0.001**	1030 ± 297.02 (700–1940)	1123.93 ± 252.26 (7.85–24.64)	** < 0.001**
Daily weight gain, g			17.48 ± 5.01 (7–31)	15.54 ± 4.81 (7.14–28.57)	19.21 ± 4.54 (9.64–31.15)	** < 0.001**	14.52 ± 5.43 (7.14–28.52)	16.19 ± 4.29 (7.85–24.64)	** < 0.001**
Days in incubator			47.5 ± 18.21 (16–117)	59.71 ± 17.02 (28–117)	36.6 ± 10.87 (16–80)	** < 0.001**	63.86 ± 19.28 (32–117)	57.04 ± 14.97 (28–94)	** < 0.001**
Oxygen Therapy, days	invasive		2.9 ± 7.20 (0–55)	5.65 ± 9.67 (0–55)	0.44 ± 1.53 (0–8)	** < 0.001**	8.81 ± 12.31 (0–55)	3.62 ± 6.90 (0–35)	** < 0.001**
	Non-invasive		17.23 ± 19.06 (0–81)	31.5 ± 18.32 (0–81)	4.49 ± 6.65 (0–49)	** < 0.001**	35.67 ± 19.22 (5–81)	28.82 ± 17.36 (0–76)	** < 0.001**
	Total		20.1 ± 22.17 (0–107)	37.09 ± 21.03 (0–107)	4.92 ± 6.90 (0–49)	** < 0.001**	44.47 ± 23.72 (5–107)	32.34 ± 17.75 (0–76)	** < 0.001**
Respiratory Distress			102 (52.3%)	69 (75%)	33 (37.5%)	** < 0.001**	28 (77.8%)	41 (73.2%)	0.621
Surfactant therapy			66 (33.8%)	48 (52.2%)	18 (17.6%)	** < 0.001**	22 (61.1%)	26 (46.4%)	0.169
Multiple pregnancy			46 (26%)	26 (28.3%)	20 (19.6%)	0.565	9 (25%)	17 (30.36%)	0.252
Patent ductus arteriosus			19 (9.7%)	15 (14.7%)	4(%3.9)	**0.016**	10 (27.8%)	5 (8.9%)	0.017

Independent Sample t-Test, Chi-Square Test

p^1^ between infants with and without ROP

p^2^ between infants with and without laser treatment

The mean GA of the entire population was 28.99 ± 2.24 weeks (range, 24–34 weeks). It was 27.61 ± 1.65 weeks (range, 24–33 weeks) in infants with ROP and 30.22 ± 1.97 weeks (range, 26–34 weeks) in infants without ROP (p < 0.001). The mean BW was 1087.17 ± 272.98 g (range, 700–1940 g) in infants with ROP and 1376.5 ± 284.47 g (range, 690–1960 g) in infants without ROP (p < 0.001). The number of days in the incubator, invasive, and non-invasive oxygen therapy were longer in infants with ROP (p < 0.001 for both). In addition, the prevalence of RDS, surfactant therapy, and PDA (p < 0.001, p < 0.001, p = 0.016) were more common in infants with ROP. Multiple pregnancy rates were similar among infants with and without ROP (p = 0.565).

In the ROP subgroups, the mean GA was 26.92 ± 1.52 (range, 24–30) weeks in infants treated with laser photocoagulation and 28.05 ± 1.59 (range, 25–33) weeks in infants not treated (p < 0.001). The mean BW was 1030 ± 297.02 g (range, 700–1940) in infants who underwent laser photocoagulation, and 1123.93 ± 252.26 (720–1940) g in infants who did not (p < 0.001). DWG was lower in the group treated with laser photocoagulation than in the group without laser treatment, and the number of days in the incubator and oxygen therapy was longer (p < 0.001). Although there were no significant differences between the two subgroups in terms of these variables (Table 1), RDS, and surfactant treatment requirements were more common in infants who had laser treatment (p = 0379, p = 0.169, respectively), and multiple pregnancy were more common in infants without laser treatment (p = 0.169).

Neutrophil, lymphocyte, and platelet counts were higher in infants with ROP on postnatal first-day than in infants without ROP (p < 0.001, p = 0.004, p = 0.008, respectively). Although the number of monocytes was higher in infants without ROP, the difference was not statistically significant (p = 0.415). NLR and SII values were significantly higher in infants with ROP than in those without ROP (p < 0.001 for both). However, the differences between the groups in terms of PLR and LMR were not significant (p = 0.93, p = 0.178, respectively). On the postnatal first-day, there was no significant difference in laboratory measurements between infants with ROP who received laser treatment and infants with ROP who did not receive laser treatment (p > 0.05). When infants with ROP were compared with infants without ROP in the postnatal first-month, it was seen that the neutrophil count was higher in infants with ROP, and the lymphocyte and platelet counts were higher in infants without ROP (p < 0.001, p = 0.023, and p = 0.019, respectively). There was no significant difference in monocyte counts between the two groups (p = 0.269). In postnatal first-month, laboratory measurements were similar between infants with ROP who had laser treatment and infants with ROP who did not have laser treatment (p > 0.05 for both) (Table 2).

**Table 2:** Laboratory measurements at postnatal first day and month among study groups

Variables	ROP group (n = 92)	Non-ROP (n = 103	P^1^ value	ROP with Laser (n = 36)	ROP without Laser (n = 56)	P^2^ value
Postnatal first day						
Neutrophil count (× 10^9^/l)	4.75 ± 2.01	3.44 ± 1.57	** < 0.001**	5.22 ± 1.87	4.45 ± 2.06	0.215
Lymphocyte count (× 10^9^/l)	4.16 ± 1.54	4.72 ± 1.33	**0.004**	4.15 ± 1.40	4.16 ± 1.64	1.000
Monocyte count (× 10^9^/l)	1.62 ± 0.67	1.76 ± 0.77	0.415	1.54 ± 0.68	1.67 ± 0.67	0.313
Platelet count (× 10^9^/l)	254.85 ± 97.47	283.07 ± 80.88	**0.008**	259.92 ± 94.87	251.59 ± 99.81	1.000
NLR	1.25 ± 0.61	0.75 ± 0.34	** < 0.001**	1.32 ± 0.49	1.21 ± 0.68	0.185
PLR	70.96 ± 42.04	63.8 ± 23.90	0.930	71.83 ± 43.47	70.39 ± 41.48	0.839
LMR	3.29 ± 2.61	3.07 ± 1.23	0.178	3.46 ± 2.38	3.18 ± 2.77	0.553
SII	328.4 ± 231.16	213.89 ± 126.76	** < 0.001**	351.02 ± 212.94	313.86 ± 242.91	0.332
Postnatal first month						
Neutrophil count (× 10^9^/l)	3.34 ± 1.56	2.55 ± 1.41	** < 0.001**	3.35 ± 1.37	3.33 ± 1.68	1.000
Lymphocyte count (× 10^9^/l)	5.48 ± 1.53	6.02 ± 1.53	**0.023**	5.25 ± 1.63	5.63 ± 1.45	0.840
Monocyte count (× 10^9^/l)	1.45 ± 0.76	1.31 ± .49	0.269	1.42 ± 0.77	1.47 ± 0.76	0.710
Platelet count (× 10^9^/l)	303.4 ± 89.05	338.71 ± 103.88	**0.019**	308.33 ± 93.53	300.23 ± 86.77	0.841
NLR	0.7 ± 0.59	0.44 ± 0.25	** < 0.001**	0.76 ± 0.73	0.66 ± 0.47	0.708
PLR	59.67 ± 24.42	59.43 ± 22.43	0.915	64.21 ± 28.71	56.75 ± 20.97	0.270
LMR	5.59 ± 5.33	4.98 ± 1.50	**0.007**	6.05 ± 7.00	5.28 ± 3.96	1.000
SII	202.64 ± 142.98	149.81 ± 109.68	** < 0.001**	209.28 ± 137.65	198.36 ± 147.37	0.488

Independent Sample t-Test

p^1^ between infants with and without ROP

p^2^ between infants with and without laser treatment

Independent variables identified as risk factors for ROP were included in univariate logistic regression analysis (Table 3). Infants with ROP had significantly lower GA (p < 0.001; OR:0.441 95% CI:0.346–0.560), BW (p < 0.001; OR:0.996 95% CI:0.995–0.998), and DWG (p < 0.001; OR:0.844, 95% CI:0.787–0.904) compared to infants without ROP. Duration in incubation (p < 0.001; OR:1.135, 95% CI:1.096–1.175), RDS (p < 0.001; OR:6.364 95% CI:3.397–11.921), and requirement for surfactant treatment (p < 0.001; OR:5.152, 95% CI:2.682–9.894) were significantly higher in infants with ROP. There was no significant difference between the two groups in terms of multiple pregnancy (p = 0.148; OR:1.635, 95% CI:0.839–3.184). Invasive, non-invasive, and total oxygen therapy was longer in infants with ROP (p < 0.001; OR:1.346, 95% CI:1.161–1.561, p < 0.001; OR:1.211, 95% CI:1.146–1.280, p < 0.001; OR:1.192, 95% CI:1.131–1.255, respectively). Postnatal first-day NLR (p < 0.001; OR:12.470, 95% CI:5.196–29.928) and SII (p < 0.001; OR:1.004, 95% CI:1.002–1.006) values were significantly higher in infants with ROP. Although the PLR and LMR values were higher in infants with ROP, no statistically significant difference was found (p = 0.145; OR:1.006, 95% CI:0.998–1.015, p = 0.453; OR:1.057, 95% CI:0.915–1.220, respectively). Postnatal first-month NLR, LMR, and SII were significantly higher in infants who developed ROP (p < 0.001; OR:10.113 95% CI:3.257–31.396, p < 0.001; OR:1.044, 95% CI:1.004–1.129, p = 0.007; OR:1.004 95% CI:1.001–1.006, respectively). The possible effects of the variables on the ROP development were studied using multivariate regression analysis (Table 4). Postnatal first-day NLR posed to highest risk for the ROP development (p = 0.002; OR:8.867 95% CI:2.275–34.564). Total oxygen therapy, postnatal first-month LMR, and RDS were also determined as high-risk factors.

**Table 3:** Univariate logistic regression analyses were performed between the groups to identify predictive factors for the development of ROP and requirements for laser photocoagulation treatment

	Development of ROP				Requirement for laser treatment			
Variables	OR	95% CI		P_1_ Value	OR	95% CI		P_2_ Value
		Lower	Upper			Lower	Upper	
Gestational age, weeks	0.441	0.346	0.560	** < 0.001**	1.653	1.200	2.278	**0.002**
Birth weight, g	0.996	0.995	0.998	** < 0.001**	1.001	1.000	1.003	0.112
Daily weight gain, g	0.844	0.787	0.904	** < 0.001**	1.079	0.984	1.184	0.106
Duration in incubation, days	1.135	1.096	1.175	** < 0.001**	0.976	0.951	1.002	0.066
Respiratory Distress Syndrome	6.364	3.397	11.921	** < 0.001**	0.781	0.292	2.088	0.622
Surfactant therapy	5.152	2.682	9.894	** < 0.001**	0.552	0.235	1.292	0.171
Multiple pregnancy	1.635	0.839	3.184	0.148	1.308	0.508	3.365	0.578
Invasive Oxygen therapy, days	1.346	1.161	1.561	** < 0.001**	0.941	0.893	0.991	**0.021**
Non-invasive Oxygen therapy, days	1.211	1.146	1.280	** < 0.001**	0.979	0.956	1.003	0.084
Total Oxygen therapy, days	1.192	1.131	1.255	** < 0.001**	0.971	0.949	0.993	**0.010**
NLR, on first day	12.470	5.196	29.928	** < 0.001**	0.736	0.370	1.464	0.383
PLR, on first day	1.006	0.998	1.015	0.145	0.999	0.989	1.009	0.872
LMR, on first day	1.057	0.915	1.220	0.453	0.960	0.819	1.126	0.620
SII, on first day	1.004	1.002	1.006	** < 0.001**	0.999	0.998	1.001	0.453
NLR, in first month	10.113	3.257	31.396	** < 0.001**	0.743	0.358	1.543	0.426
PLR, in first month	1.000	0.988	1.013	0.944	0.987	0.970	1.005	0.165
LMR, in first month	1.044	1.004	1.129	** < 0.001**	0.974	0.901	1.053	0.502
SII, in first month	1.004	1.001	1.006	**0.007**	0.999	0.997	1.002	0.720

Binary Logistic Regression Analysis

P_1_: Between groups with and without ROP to identify predictive factors for the development of ROP

P_2_: To detect predictive factors for laser treatment in infants with ROP

**Table 4:** Multivariate logistic regression analyses for ROP development and requirement for laser treatment

Variables	OR	95% CI		P Value
		Lower	Upper	
Development of ROP				
Respiratory Distress Syndrome	3.524	1.140	10.894	**0.029**
Total Oxygen therapy, days	1.184	1.121	1.250	** < 0.001**
NLR, on first day	8.867	2.275	34.564	**0.002**
LMR, in first month	1.286	1.064	1.555	**0.009**
Requirement for laser treatment				
Gestational age, weeks	1.702	1.135	2.552	**0.010**
Non-invasive Oxygen therapy, days	1.091	1.014	1.174	**0.020**
Total Oxygen therapy, days	0.902	0.837	0.972	**0.007**
PLR, postnatal first month	0.951	0.919	0.984	**0.004**
SII, postnatal first month	1.011	1.004	1.018	**0.004**

Multiple Logistic Regression Analysis

**Fig. 1:**
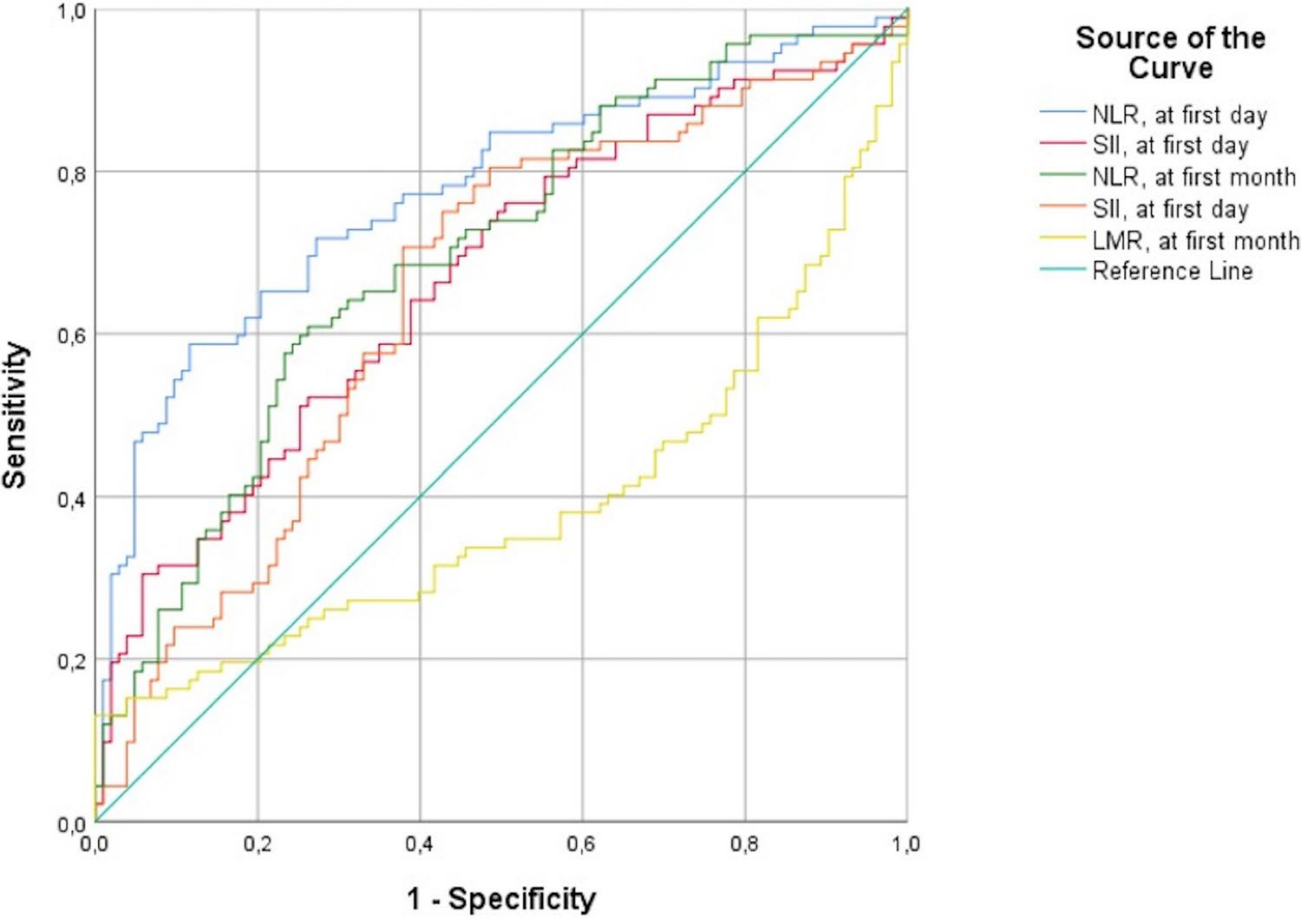
ROC curve analysis of laboratory parameters for ROP development

Figure 1 shows the sensitivity and specificity of NLR and SII on postnatal first-day and NLR, SII, and LMR at postnatal first-month and the area under the ROC curve as risk factors for the ROP development. Postnatal first-day NLR of 1.05 or higher predicted the ROP development with 58.7% sensitivity and 88.3% specificity, the area under the curve was 0.774 (p < 0.001; 95% CI: 0.707–0.841). Postnatal first-day SII value of 254.98 or higher was seen as a predictor for the ROP development with 52.17% sensitivity and 73.8% specificity, and the area under the curve was 0.670 (p < 0.001; 95% CI: 0.594–0.746). The area under the curve for NLR at the postnatal first-month was 0.698, and the NLR value of 0.51 or higher predicted the development of ROP with a sensitivity of 60.87% and a specificity of 73.8% (p < 0.001; 95% CI: 0.625–0.772). Postnatal first-month SII value of 133.84 or more was considered a risk factor for the ROP development with 70.65% sensitivity and 62.1% specificity (p < 0.001; 95% CI:0.572–0.728), and the area under the curve was 0.650. Postnatal first-month LMR value of 3.84 or higher was regarded as a risk factor for the development of ROP with 50% sensitivity and 77.1% specificity, the area under the ROC curve was 0.612 (p < 0.007; 95% CI: 0.529–0.695).

We examined the risk factors for the requirement of laser treatment in patients with ROP by using univariate logistic regression analysis (Table 3). GA was significantly lower in infants who underwent laser treatment (p = 0.02; OR:1.653; 95% CI: 1.200–2.278). Invasive and total oxygen therapy was longer in infants who received laser treatment (p = 0.021; OR:0.941; 95% CI:0.893–0.911, p = 0.010; OR:0.971; 95% CI:0.949–0.943, respectively). No significant differences were found for other risk factors. Multivariate analysis was run to predict the variables affecting laser treatment requirements (Table 4). Lower GA and longer total and noninvasive oxygen therapy duration were found as predictors of laser treatment requirements in infants with ROP (p = 0.010; OR:1.702; 95% CI: 1.135–2.552, p = 0.007; OR: 0.902; 95% CI: 0.837–0.972, p = 0.020; OR: 1.091; 95% CI: 1.014–1.1724, respectively). In multivariate model, postnatal first-month SII and PLR values were identified as independent predictors for laser treatment requirements. (p = 0.004; OR:1.011; 95% CI:1.004–1.018, p = 0.004; OR:0.951; 95% CI:0.919–0.984, respectively). The GA is the most significant predictor of laser treatment requirement in infants with ROP.

## Discussion

Neonatal inflammation plays an active role in ROP pathogenesis [6]. Systemic inflammation due to cytokines is associated with the risk of ROP development [20]. In recent years, the NLR, LMR, PLR, and SII have been frequently reported as markers of inflammation in both ophthalmology and systemic diseases [11, 12]. However, limited number of studies have focused on the relationship between SII and ROP [17]. In this study, we investigated the effects of inflammatory parameters for ROP development and the need for treatment. In our study, postnatal first-day NLR and SII values and postnatal first-month NLR, SII, and LMR values were significantly higher in terms of ROP development. PLR values were not significantly different among infants with and without ROP on either postnatal first-day or first-month.

In our study, we found that neutrophil counts in infants with ROP and lymphocyte and platelet counts in infants without ROP were significantly higher on both postnatal first-day and first-month. Monocyte count was similar in all patients. It is a well-known fact that there will be a decrease in the number of neutrophils and an increase in the number of lymphocytes after birth [21]. We also observed a numerical change in our patients; however, this statistical difference was still obvious in the postnatal first-month. Neutrophils are indicators of systemic inflammation, lymphocytes are indicators of physiological stress, and their balance reflects immune responses [22]. NLR shows this immune balance. In our study, NLR values at both postnatal first-day and first-month were determined as risk factors for the development of ROP. While postnatal first-day and first-month NLR values were associated with the ROP development in univariate analysis, the postnatal first-day NLR value pointed to the parameter showing the highest risk for ROP development. However, NLR values at both postnatal first-day and first-month were not risk factors for ROP treatment requirement. In the study of Kurtul et al., similar to our study, the NLR value in the complete blood count at birth was significantly higher in infants with ROP than in infants without ROP [23]. However, it lost its significance in multivariate analysis [23]. This difference between the two studies might be due to the limited number of patients in the control group in the study conducted by Kurtul et al. [23]. In another study, the NLR value was higher in infants without ROP compared to infants with ROP, which is inconsistent with our study given the CBC calculated the day after birth [24]. Some of the infants included in this study had neonatal pneumonia [24]. We contend that this situation might have affected the complete blood count parameters. In a recent study, it was reported that the NLR value was not associated with the ROP development [15]. However, the same study stressed that high NLR values were associated with the need for laser therapy in infants with ROP [15]. This might be attributed to the fact that infants with ROP treatment are often in worse general condition than infants without ROP treatment [15]. We found no relationship between NLR values and ROP treatment requirements. The difference between our study and the findings of the study explained above could stem from the fact that infants with a diagnosis of sepsis were not excluded from the study by Obata et al. [15]. As a result of our study, we found that the postnatal first-day and first-month NLR values were associated with the ROP development. The cut-off value of NLR was calculated for the development of ROP by constructing a ROC curve. NLR values higher than 1.05 on postnatal first-day and 0.51 at postnatal first-month were associated with a high risk of ROP development.

Although an obvious relationship between low platelet count and the development of ROP has not been substantiated, some studies have suggested that low platelet count leads to the development of ROP [25, 26]. Platelets contain pro-angiogenic (such as vascular-endothelial growth factor and insulin-like growth factor-1) and anti-angiogenic (such as endostatin) regulators [27]. Activation or inhibition of angiogenesis occurs when these molecules are activated under different conditions [27]. In our study, we found that the platelet count was significantly lower in infants who developed ROP on postnatal first-day and first-month compared to those who did not develop ROP. Keşkek et al. reported that a low platelet count measured in the first week after birth was an independent risk factor for the development of ROP [28]. In a study by Tao et al., platelet counts were similar at birth among infants who developed ROP and infants who did not develop ROP, while they were lower in infants who developed ROP during the first month [29]. The results of these two studies were similar to those of our study in terms of the effect of platelet count on the development of ROP. We did not detect any effect of the platelet count on the need for laser photocoagulation treatment. In our study, PLR was higher in infants with ROP on the postnatal first-day, and it was at a similar level in the postnatal first-month. There were no significant differences between the two groups in either value. In the study of Hu et al., although PLR was found higher in infants with ROP than in infants without ROP on the day after birth, there was no statistical difference [24], and the findings of our study corroborates these findings. In our study, PLR was not a risk factor for laser photocoagulation.

In our study, the postnatal first-month LMR value was significantly higher in infants with ROP than in those without ROP. In the logistic regression analyses, the LMR value measured in postnatal first-month was determined as an independent risk factor for the ROP development. In the study of Hu et al., postnatal first-day LMR value was reported as an independent risk factor for ROP development [24]. However, postnatal first-day LMR values were not observed as a risk factor in our study. In the aforementioned study, the rate of neonatal pneumonia was higher in patients with ROP than in patients without ROP [24]. This may explain the inconsistency between the two studies in terms of postnatal first-day LMR values as a risk factor. In our study, ROC analysis was performed to calculate the cut-off value of postnatal LMR in predicting the development of ROP. Accordingly, the cut-off value was 3.84. In the study of Hu et al., the cut-off value determined immediately after birth was 3.21; to wit, approximately equal to the value we found [24]. We did not detect any effects of LMR value on the need for laser treatment in infants with ROP using logistic regression analyses.

SII is a parameter that refers to the immune balance between platelet, neutrophil, and lymphocyte counts, the frequency of which has been increasing in recent years [30–32]. Nonetheless, limited number of studies have examined the relationship between ROP development and SII [17]. In a study by Akdoğan et al., while the SII value detected on the day after birth did not show a significant difference between infants with and without ROP, the SII value found at postnatal first-month was reported to be significantly higher in infants with ROP [17]. Similarly, in our study, postnatal first-month SII values were significantly higher in patients with ROP. However, in our study, unlike Akdoğan et al., we found that postnatal first-day SII values were significantly higher in infants with ROP [17]. We created a ROC curve to determine the cut-off value of postnatal first-day and first-month SII values. We found that when the SII value on postnatal first-day is over 254.98 and above 133.84 in the postnatal first-month, it poses a risk for the development of ROP. However, we did not find any significant differences in terms of ROP development in multivariate analyses. When the need for laser photocoagulation and SII were taken into consideration in our study, we found that the SII value in multivariate analysis was an independent risk factor for the need for laser photocoagulation. To the best of our knowledge, our study is the first to examine the relationship between laser treatment needs and SII.

Despite our best endeavors, we should acknowledge that our study has several limitations. Firstly, it is a retrospective study. Secondly, our study lacks other inflammation parameters, such as CRP. Therefore, further prospective studies with a larger number of patients are warrented to examine the parameters of inflammation.

In this study, we probed the relationship between inflammation and the development of ROP and other requirements for laser treatment. Based on the findings, the postnatal first-day NLR and postnatal first-month LMR values were independent risk factors for the development of ROP in multivariate analyses. The parameter indicating the highest risk for ROP development was the postnatal first-day NLR values. Postnatal first-month PLR and SII values in the first-month were independent risk factors for laser treatment.

## Declarations

The institution at which the study was conducted: Başkent University School of Medicine Department of Ophthalmology, Adana Hospital.
